# Innate positive chemotaxis to paeonal from highly attractive Chinese medicinal herbs in the cigarette beetle, *Lasioderma serricorne*

**DOI:** 10.1038/s41598-019-43198-3

**Published:** 2019-05-06

**Authors:** Yu Cao, Giovanni Benelli, Giacinto Salvatore Germinara, Filippo Maggi, Yuanjie Zhang, Shuangli Luo, Hong Yang, Can Li

**Affiliations:** 1 Guizhou Provincial Key Laboratory for Rare Animal and Economic Insect of the Mountainous Region, Department of Biology and Engineering of Environment, Guiyang University, Guiyang, Guizhou 550005 P.R. China; 2 Department of Agriculture, Food and Environment, University of Pisa, via del Borghetto 80, 56124 Pisa, Italy; 3 Department of the Sciences of Agriculture, Food and Environment, University of Foggia, Via Napoli 25, Foggia, 71122 Italy; 4 School of Pharmacy, University of Camerino, Via Sant’Agostino 1, 62032 Camerino, Italy

**Keywords:** Entomology, Behavioural ecology

## Abstract

*Lasioderma serricorne*, also known as cigarette beetle, can exploit a wide variety of stored materials as foods, but it is particularly common on tobacco and herbs. This beetle is a dominant pest species of stored Chinese medicinal materials (CMMs) causing high economic damages, making effective control strategies urgently needed. Behavioural manipulation is an important component of Integrated Pest Management. To the best of our knowledge, plant-borne volatile organic compounds (VOCs) have never been explored to develop lures for managing *L*. *serricorne*. In this study, the behavioural responses of *L*. *serricorne* to VOCs from four selected CMMs (*Euphorbia kansui*, *Aconitum carmichaelii*, *Eucommia ulmoides* and *Pinellia ternata*) were studied and their components analysed. Then, the olfactory responses of *L*. *serricorne* to the most abundant VOC identified in the preferred CMM, i.e., paeonal, was tested. *L*. *serricorne* showed significant differences in its preferences for the VOCs from the four CMMs, i.e, *E*. *kansui* > *A*. *carmichaelii* > *E*. *ulmoides* > *P*. *ternata*. From the VOCs of *E*. *kansui*, *A*. *carmichaelii*, *E*. *ulmoides*, and *P*. *ternata*, 77, 74, 56, and 81 molecules, were identified, respectively. Paeonal (23.5%), junipene (17.2%), hexanal (17.1%), and benzeneacetonitrile (14.0%) were the most abundant, respectively. Since paeonal dominated the VOC spectrum of the most preferred CMM, this compound was selected for further studies. *L*. *serricorne* showed significant positive responses to paeonal tested at various doses, with the most attractive ones being 100 μg and 500 μg. Our findings shed light on the olfactory cues routing the food searching behaviour in the cigarette beetle, providing important information on how *L*. *serricorne* targets particular CMMs. The high attractiveness of paeonal at low doses tested here may be exploited further to develop novel monitoring and control tools (e.g., lure-and-kill strategies) against this important stored product pest.

## Introduction

The cigarette beetle, *Lasioderma serricorne* Fabricius (Coleoptera: Anobiidae), was first reported on tobacco in France in 1848^1^. This pest occurs frequently in tropical and subtropical areas, and is now distributed worldwide^2^. *L*. *serricorne* consumes a wide range of dry foods and currently ranks as one of the most serious pests of stored products worldwide^3–5^. Consequently, it causes huge damages and economic losses to post-harvest and stored grains and seeds, packaged food products, including many animal- and plant-derived products^6^. It is especially injurious to stored tobacco^7^.

Pesticides, such as phosphine, have been widely used against insects on stored products for more than 100 years, but some serious negative issues, including insecticide resistance and lethal effects on non-target organisms, have resulted owing to their repeated and overzealous use^8–11^. In addition, the abuse or intensive use of synthetic chemical pesticides has driven some species to the edge of, or into, extinction^12^. The negative ecological and health impacts have attracted research and public opinion attention^9^. Therefore, available alternatives to chemical insecticides against stored-product pests are urgently required^13–18^.

There is a long history of using botanical insecticides to protect stored products from insect pests^19,20^. Various repellents and attractants, which have functional components derived from specific plants, have been applied to manipulate insect behaviour, effectively protecting stored products from pest infestation^14,21,22^. Thus, there is a keen interest in the development of botanical pesticides^23,24^.

Behavioural manipulation is an important and eco-friendly insect control method aimed to exploit behavioural responses of insects to selected cues from the environment, to manage pest populations^21^. Host-plant volatiles can significantly influence host location behaviour of insects^25^. For stored-product beetles, several pest species, including *Sitophilus granarius* Linnaeus (Curculionidae), *S*. *zeamais* Motschulsky (Curculionidae), *Oryzaephilus surinamensis* Linnaeus (Sylvanidae) and *Callosobruchus maculatus* Fabricius (Bruchidae) are attracted to the volatiles of wheat, peas, maize, chickpeas, or other stored food products^26–29^. Therefore, it is reasonable to attempt developing effective attractants or repellents based on the components of mixtures of volatile organic compounds (VOCs) from specific stored products, which could be used for the management or behavioural manipulation of stored-product pests.

Nowadays, several cues attractive to *L*. *serricorne* have been investigated, attempting various monitoring and control approaches^30^. About physical stimuli, both sexes are attracted by UV lights, therefore electric traps have been used^31–33^. Concerning olfactory ones, female pheromones, i.e., anhydroserricornin 2,6-diethyl-3,5-dimethyl-3,4-dihydro-2H-pyran^34^ and serricornin 4,6-dimethyl-7-hydroxynonan-3-one as 4S, 6S, 7S^35,36^, have been synthesized and used to as trap lures for pest monitoring^37–41^. From a real-world perspective, commercial traps (commonly known as Lasiotrap) and specially designed multisurface traps have been successfully proposed for mass-trapping and delaying cigarette beetle infestation in tobacco stores^42^.

Despite the rather wide knowledge about the light cues and sex pheromones attractive to *L*. *serricorne* beetles, information on the plant-borne VOCs potentially useful to improve monitoring and mass-trapping tools remains limited. Notably, even if adult females scarcely feed^43,44^, it has been stressed that they are attracted by foods suitable for their oviposition^45^. Later, Papadopoulou and Buchelos pointed out that tablets containing herbal extracts can be placed in traps to boost their attractiveness^30^. However, the composition of herbal extracts is often hard to standardize from a chemical point of view, due to the large phytochemical variations linked with the plant geographical origin, growing conditions and harvesting time^46,47^. Thus, assessing the effectiveness of pure compounds isolated from botanical products is important for real-world applications. In this scenario, one may argue: what do we really know about the attraction of cigarette beetles to VOCs from stored Chinese medicinal materials (CMMs)? Are selected pure compounds from CMM VOC bouquets attractive to them? Do they show a dose-dependent bioactivity?

Indeed, *L*. *serricorne* is usually found in economically important stored products, such as tobacco and traditional CMMs^48,49^. In China, it causes major losses to the CMMs industry during storage in Guizhou Province^49^. Notably, the damage levels vary among the different CMMs, indicating a certain host preference for this pest. However, there is strictly limited information on the behavioral responses of *L*. *serricorne* to odors from CMMs.

*Euphorbia kansui* T.N.Liou ex T.P.Wang (Euphorbiales: Euphorbiaceae), *Aconitum carmichaelii* Debeaux (Ranales: Ranunculaceae), *Eucommia ulmoides* Oliver (Garryales: Eucommiaceae), and *Pinellia ternata* Breitenbach (Arales: Araceae) are four widely planted CMMs of important economic value in China that are damaged, to different degrees, by *L*. *serricorne*. However, to the best of our knowledge, CMMs-borne olfactory cues routing food location in *L*. *serricorne* have been scarcely studied.

Therefore, in this research, to explore the importance of chemical cues routing *L*. *serricorne* host preference, the olfactory responses of *L*. *serricorne* adults to VOCs emitted from the four CMMs reported above were studied in Y-tube olfactometer bioassays. Furthermore, to develop potential novel lures to be used against *L*. *serricorne* for monitoring and control purposes, VOCs from these CMMs and their relative abundances were analyzed by headspace solid-phase microextraction (SPME) coupled with gas chromatography–mass spectrometry (GC–MS), and then the behavioural responses of *L*. *serricorne* to the most abundant compound - identified from the most preferred VOC spectrum of CMMs - were tested in Y-tube and six-arm olfactometer assays.

## Materials and Methods

### Insects rearing

*Lasioderma serricorne* was reared in our laboratory since 2015, maintaining the beetles on the CMMs *Angelica sinensis* Oliver (Apiales: Apiaceae). Cigarette beetles were reared at 27 ± 1 °C, with 55 ± 5% R.H. under 8:16 (L:D) photoperiod (photophase between 9:00 a.m. and 5:00 p.m.) as described by Li *et al*.^49^. These conditions prevented secondary infestations by moisture-sensitive mites^50^.

### Behavioral responses of *L*. *serricorne* to CMM-borne volatiles

#### Odor sources

CMMs *E*. *kansui*, *A*. *carmichaelii*, *E*. *ulmoides*, and *P*. *ternata* (moisture content 11–15%, 11–15%, 8–11%, and 12–14%, respectively) were purchased from the Chinese Traditional Medicine Market (Guiyang City, Guizhou Province, China), and stored free of pesticides or pest infestations. As the main medicinal parts differed from the four CMMs and were stored for medical treatment, the roots from *E*. *kansui* and *A*. *carmichaelii*, barks from *E*. *ulmoides* and tubers from *P*. *ternata* were used for experiments in this study.

#### Y-tube bioassays

The olfactory responses of *L*. *serricorne* were tested in a Y-tube olfactometer (stem 195 mm long, each arm 130 mm long at a 135° angle, all 15-mm i.d.) using the method recently described by Ndomo-Moualeu *et al*.^29^ and Cao *et al*.^51^. Two types of two-way comparisons were made: (1) each CMMs (25 g) *versus* clean air (CA); and (2) all possible CMM pairings (25 g each). Unmated adult *L*. *serricorne* (2–3 days old) were used in these experiments. Air flow was set as 200 mL/min/arm, the air flow was passing through activated charcoal (for purification) and distilled water (for humidification) before entering in each odour chamber and then in each arm of the Y-tube^51^. In total, 50 adults were used to test each odour treatment, with the olfactometer cleaned after each tested beetle, as described by Carpita *et al*.^52^. The arm positions were reversed to avoid positional bias after 5 individuals were tested, and the Y-tube olfactometer was replaced with a fresh one after 10 beetles were tested. All bioassays were conducted between 9:00 a.m. and 5:00 p.m.

To understand whether there was any intrinsic bias within the olfactometer, 24 runs were carried out with CA passing through each empty chamber and with unmated insects, 24 different *L*. *serricorne* adults (2–3 days old) were tested individually. The time required for the insect to make a choice was recorded. If the beetle had not made a choice after 30 min, another beetle was tested. The longest time taken for all the responding insects was recorded and used as the maximum run time in subsequent tests.

To understand whether *L*. *serricorne* could distinguish between odour stimuli and CA, 30 runs were carried out with 25 g of *A*. *sinensis* (feeding materials used for rearing *L*. *serricorne*) in one branch and CA in the other branch. Equal numbers of male and female beetles were tested.

### Collection and analysis of volatiles

The collection and analysis of CMM VOCs were conducted as recently detailed by Cao *et al*.^51^. Different CMMs (each 2.0 g) were placed in glass bottles for 2 h to confine the odour, and then headspace volatiles were collected using a solid-phase microextraction (SPME) fiber. A manual injector and a ~50/30 µm DVB/CAR/PDMS StableFlex fiber head was inserted into the mouth of the bottle. VOCs were extracted for 40 min at 80 °C, then the fibre head was quickly removed and inserted into the injection port of the gas chromatograph (GC) (250 °C, run in splitless sampling mode). The collected VOCs were analyzed by GC–MS (HP6890/5975 C, Agilent Technologies). An apolar chromatographic column (ZB-5MSI 5% phenyl-95% dimethylpolysiloxane 30 m × 0.25 mm, film thickness 0.25 μm) was used. Temperature was programmed to rise from 40 to 255 °C with a 5 °C/min rate, and then maintained for 2 min. The temperatures of the vaporizing chamber, interface, and quadrupole rod were set at 250 °C, 280 °C, and 150 °C, respectively. The chemical identities of the peaks that were mainly present in CMMs were determined by comparing the mass spectra of compounds with those in databases (NIST 2017 and WILEY 275). Moreover, the coherence of the temperature-programmed retention indices (RIs) with respect to those recorded in Adams^53^ and NIST 17^54^ was used as an additional criterion for peak assignment. Spectra and retention times were also compared with those of authentic standards, which were purchased from Sigma-Aldrich, Germany. However, the tentative identification of compounds was not confirmed in this study. Additional parameters were as follows: delay time of solvent, 1.0 min; ion source, EI; ionization potential, 70 eV; emission current, 34.6 μA; voltage of the multiplier, 1671 V; and scanning from 29 to 500 atomic mass units.

### Behavioural responses of *L*. *serricorne* to paeonal from *E*. *kansui* VOCs

The VOC mixture from *E*. *kansui* was the most attractive to *L*. *serricorne*, as assessed by the Y-tube olfactometer bioassays. Therefore, since paeonal was the most abundant compound identified from the VOCs of *E*. *kansui*, the behavioral responses of *L*. *serricorne* to paeonal were tested as follows.

#### Odour treatment

Paeonal (Sigma-Aldrich, Germany; chemical purity 99%) solutions (i.e., 0.1, 1, 10, 50 and 100 μg μl^−1^) were prepared with mineral oil (Sigma-Aldrich, Germany). Solutions were stored at −20 °C until the testing phase.

#### Y-tube bioassays

The Y-tube olfactometer described above was also used here to test the olfactory responses of *L*. *serricorne* to paeonal. Mineral oil was used as a control, a test (at the concentrations detailed above, ranging from 0.1 to 100 μg μl^−1^) or control (10 μl of paeonal solution or mineral oil, respectively) stimulus was adsorbed onto a filter paper disk (1.0-cm diameter) placed in the odor chamber. *L*. *serricorne* beetles can choose between a specific dose of the tested stimulus (1, 10, 100, 500, and 1000 μg) and the mineral oil. Bioassays were conducted using the method detailed above; 2–3 days old unmated *L*. *serricorne* adults were tested. In total, 50 adults were tested per each cue and compared with mineral oil.

#### Six-arm olfactometer bioassays

The behavioural responses of adult *L*. *serricorne* to different doses of paeonal were also evaluated in a six-arm olfactometer in agreement with the method reported in Liu *et al*.^55^. Briefly, the six-arm olfactometer consisted of a central chamber (12-cm internal diameter) with six arms (6-cm length and 1.5-cm internal diameter), each connected to a glass tube (20-cm length and 1.5-cm diameter) that projected outwards at an equidistance, with 60° angles between pairs of tubes. Each arm was connected through Teflon tubing to a glass vessel, which was used to contain a test or control stimulus (10 μl). Paeonal doses of 1, 10, 100, 500, and 1000 μg were used as test stimuli, and the airflow was set at 200 ml/min to drive the odour source to insects. *L*. *serricorne* (2–3 days old adults after emergence that were starved for 3 h) were introduced in groups (150 individuals per group) into the central chamber with a brush. *L*. *serricorne* that entered an arm of the olfactometer within 20 min were counted as having made a choice for a particular odour source. The beetles that did not enter an arm within this time were considered ‘non-responders’. After each test, the olfactometer was cleaned, dried and the arms were rotated (60°). Bioassays were replicated six times and were carried out between 9:00 a.m. and 5:00 p.m. A 25-W light was also placed in the centre, 60 cm above the chamber to eliminate any light bias.

### Statistical analysis

The null hypothesis that *L*. *serricorne* adults showed no preference for either Y-tube arm (a response equal to 50:50) was analysed using a chi-square goodness-of-fit test. Choices made by male and female *L*. *serricorne* were examined separately and, if there was no significant difference in the variances between the male and female beetles, then the data were pooled, and the choices were considered, regardless of sex^51,56^. The number of insects found in the different arms of the six-arm olfactometer were subjected to Friedman two-way ANOVA by ranks and in the case of significance (*P* < 0.05) the Wilcoxon signed ranks test was used for separation of means. All statistical analyses were performed using SPSS 18.0 for Windows (SPSS Inc., Chicago, IL, USA).

### Human and animal rights

This research did not involve any human participants and/or animals, only the cigarette beetle, *L*. *serricorne*.

## Results

### Behavioural responses of *L*. *serricorne* to CMMs-borne volatiles

#### Y-tube bioassays

When *L*. *serricorne* were tested for their relative preferences between the two arms of the Y-tube olfactometer linked to chambers with CA, there was no bias in a series of 24 experiments; 12 beetles chose each arm. The mean time for a beetle to make a choice was 132.3 ± 10.7 s, and the maximum time was 231 s. When *L*. *serricorne* were presented to *A*. *sinensis* versus CA, 21 females chose *A*. *sinensis* and 9 CA, while 19 males chose *A*. *sinensis* and 11 CA. Because there was no difference in the response to *A*. *sinensis* between male and female *L*. *serricorne*, these results were pooled. *L*. *serricorne* significantly preferred *A*. *sinensis* (40) to CA (20) (*χ*^2^ = 6.67, *df* = 1, *P* = 0.01). The mean time for *L*. *serricorne* to make a choice was 103.0 ± 6.1 s, and the maximum time was 223 s. Therefore, *L*. *serricorne* could distinguish between the CMMs odours and CA, and the maximum run time was 231 s. Thus, the responses of *L*. *serricorne* to volatiles were observed for 240 s (4 min) in the Y-tube olfactometer in following test.

**Figure 1:**
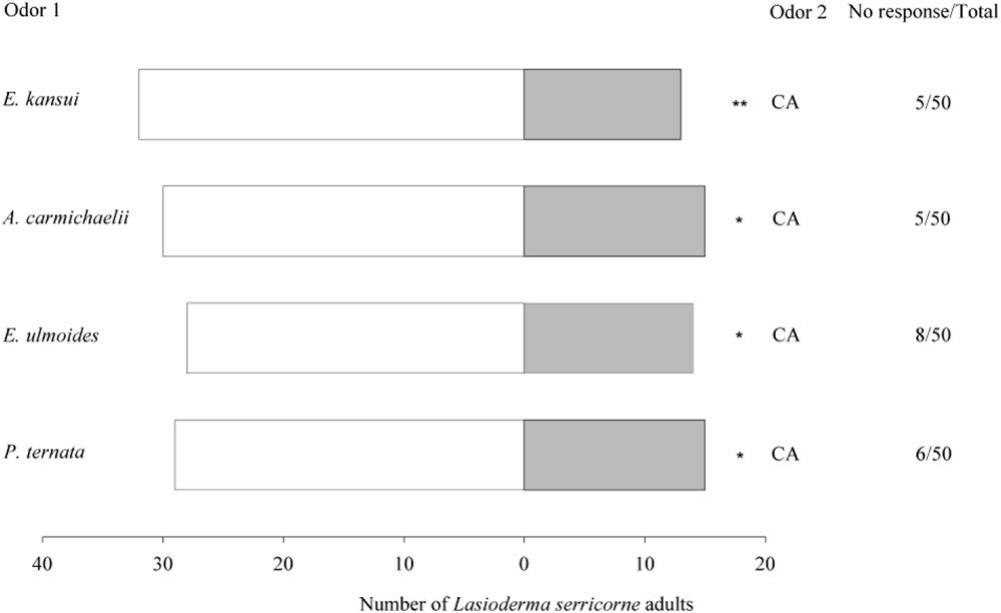
Olfactory responses of *L*. *serricorne* to odours of Chinese medicinal materials (CMMs) versus clean air (CA) in a Y-tube olfactometer. Significant differences: *P < 0.05 and **P < 0.01 (chi-square test).

*L*. *serricorne* showed significant relative preferences for *E*. *kansui* (*χ*^2^ = 8.022, *df = *1, *P* = 0.005), *A*. *carmichaelii* (*χ*^2^ = 5.000, *df* = 1, *P* = 0.025), *E*. *ulmoides* (*χ*^2^ = 4.667, *df = *1, *P* = 0.031), and *P*. *ternata* (*χ*^2^ = 5.233, *df* = 1, *P* = 0.035), when compared with CA (Fig. 1).

**Figure 2:**
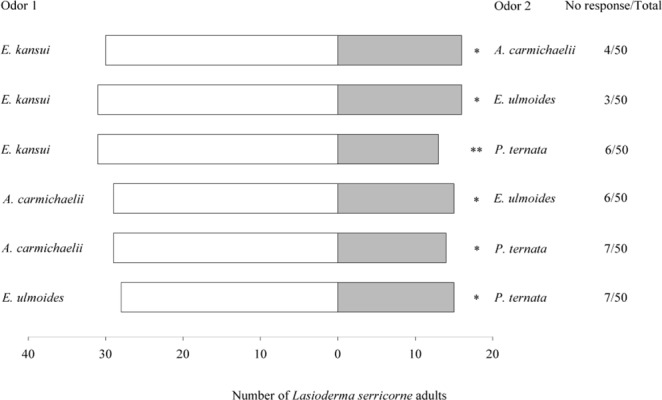
Olfactory responses of *L*. *serricorne* to odour pairings of different Chinese medicinal materials (CMMs) in Y-tube olfactometer tests. Significant differences: *P < 0.05 and **P < 0.01 (chi-square test).

When CMMs volatiles were compared each other, *L*. *serricorne* adults significantly preferred *E*. *kansui* to *A*. *carmichaelii* (*χ*^2^ = 4.261, *df* = 1, *P* = 0.039), *E*. *kansui* to *E*. *ulmoides* (*χ*^2^ = 4.787, *df* = 1, *P* = 0.029), *E*. *kansui* to *P*. *ternata* (*χ*^2^ = 7.043, *df* = 1, *P* = 0.008), *A*. *carmichaelii* to *E*. *ulmoides* (*χ*^2^ = 5.233, *df* = 1, *P* = 0.035), *A*. *carmichaelii* to *P*. *ternata* (*χ*^2^ = 5.233, *df* = 1, *P* = 0.022), and *E*. *ulmoides* to *P*. *ternata* (*χ*^2^ = 3.930, *df* = 1, *P* = 0.047) (Fig. 2).

### Analysis of volatiles

We identified 77 components in the volatile fraction of *E*. *kansui* (Table 1). The component with the highest relative content was paeonal (23.5%), followed by 3,5-dimethoxytoluene (6.5%) and safrole (5.0%), while none of the other volatiles were present at a greater than 5% content.

**Table 1:** VOCs identified from the four Chinese medical materials analysed in this study. Values (%) indicated the mean peak area for each compound estimated during GC-MS analyses.

Compounds^a^	Class^b^	Retention index (RI)^c^	Literature RI (ADAMS/NIST17)^d^	*E*. *kansui* (%)	*A*. *carmichaelii* (%)	*E*. *ulmoides* (%)	*P*. *ternata* (%)
Acetaldehyde	ALD	404	412		0.1		3.5
Formic acid	FA	518	526			1.1	
2-Methylpropanal	ALD	550	554				0.1
Butanal	ALD	588	593			0.2	0.1
2,3-Butanedione	KE	591	594	0.1			
2-Methylfuran	FUR	602	606			0.1	
Acetic acid	FA	610	606	4.1	3.5	2.0	2.8
2-Butenal	ALD	620	629			0.3	
3-Methylbutanal	ALD	647	651	0.1			0.1
2-Methylbutanal	ALD	660	663				0.1
2-Methyl-4,5-dihydrofuran	FUR	670	662			0.1	
1-Penten-3-one	KE	681	680			1.4	
1-Penten-3-ol	ALC	684	686			0.2	
2-Pentanone	KE	688	682			0.2	
Pentanal	ALD	699	704	0.1			0.6
2-Ethylfuran	FUR	701	704			0.3	
(2*E*)-Penten-1-al	ALD	754	744			0.4	
1-Pentanol	ALC	760	765	0.1		1.5	0.2
1,3-Butanediol	ALC	780	785	1.2	0.9		0.2
2,3-Butanediol	ALC	788		1.4	0.9		0.2
Hexanal	ALD	793	801	2.3		17.1	13.1
Furfural	FUR	833	828		0.1		0.1
(2*E*)-Hexenal	ALD	854	846	0.1		0.4	
2-Methylbutyric acid	FA	858	862		0.1		
3-Methylbutyric acid	FA	863	867		0.2		
1-Hexanol	ALC	868	872	0.1	0.1	2.1	0.1
2-Heptanone	KE	891	889	0.2		0.4	0.1
2-Butylfuran	FUR	892	894	0.2			
Cyclohexanone	KE	894	896				0.6
Heptanal	ALD	897	901	0.4	0.1	1.7	0.7
(2*E*,4*E*)-Hexadienal	ALD	911	907			0.4	
Butyrolactone	LACT	915	915	0.5	0.1		0.1
2,6-Dimethylpyrazine	NITR	916	919				0.4
Methyl caproate	EST	920	924			0.5	
(2*E*)-Heptenal	ALD	950	957	0.2	0.1	0.8	0.2
Benzaldehyde	ARO	962	952	0.2	0.1		1.3
5-Methyl furfural	FUR	969	964		0.1		
1-Heptanol	ALC	976	970			0.6	
Hexanoic acid	FA	978	967		1.1	2.8	2.2
1-Octen-3-one	KE	979	980			0.1	0.1
1-Octen-3-ol	ALC	980	974	0.2		1.0	0.8
2,3-Octanedione	KE	984	986				0.5
6-Methyl-5-hepten-2-one	KE	988	986	0.4	0.3		0.4
Yomogi alcohol	MO	990	999	1.0			
2-Amylfuran	FUR	998	990	0.3	0.1	1.0	1.4
Octanal	ALD	1003	998		0.3	5.2	0.7
(2*E*,4*E*)-Heptadienal	ALD	1012	1005	0.1			
(2*E*,4*E*)-Heptadienal	ALD	1012	1005			0.5	
*p*-Cymene	MH	1025	1020	0.1	0.1	0.4	
Limonene	MH	1030	1024	0.3	0.2	1.3	
2-Ethylhexanol	ALC	1030	1031		0.1		0.3
1,8-Cineole	MO	1032	1025	0.3			
Benzenemethanol	ARO	1036	1035	0.3	0.1		
(*Z*)-β-Ocimene	MH	1038	1032		0.1		
3-Octen-2-one	KE	1040	1042				1.1
Benzene acetaldehyde	ARO	1045	1036	0.1	0.1		0.4
(*E*)-β-Ocimene	MH	1049	1044	0.1			0.1
(2*E*)-Octen-1-al	ALD	1060	1059	0.3	0.1	4.7	0.2
2-Acetylpyrrole	NITR	1064	1063		0.1		
Acetophenone	ARO	1065	1059		0.1		
1-Octanol	ALC	1071	1063		0.2	1.1	0.2
Artemisia alcohol	MO	1072	1080	1.2			
(*E*,*E*)-3,5-Octadien-2-one	KE	1073	1072	0.7		0.6	0.4
*p*-Cresol	ARO	1075	1079				0.1
Tetramethylpyrazine	NITR	1089	1087	0.5			
Linalool	MO	1099	1095	0.3	0.2		0.1
Undecane	ALK	1100	1100	0.1			
Nonanal	ALD	1112	1100	2.4	1.3	11.0	5.3
Maltol	PYR	1113	1106	0.1			
Benzeneethanol	ARO	1119	1116	0.2	0.2		
Camphor	MO	1145	1141	1.1	1.0		1.1
Benzeneacetonitrile	NITR	1149	1142		0.2		14.0
Menthone	MO	1155	1148	0.1			
Pentylbenzene	ARO	1158	1160		0.3		
(2*E*)-Nonen-1-al	ALD	1164	1157	0.4	0.3	1.1	1.5
Borneol	MO	1168	1165	0.5	0.3		0.1
Benzoic acid	ARO	1170	1172				0.2
Menthol	MO	1174	1167		0.4		0.3
Terpinene-4-ol	MO	1181	1176	1.2	1.9		0.3
α-Terpineol	MO	1192	1186	0.7	4.5		
2-Decanone	KE	1199	1193			0.2	
Dodecane	ALK	1200	1200	0.3	0.2		0.3
Decanal	ALD	1206	1201	1.1	0.7	3.0	3.1
Octanol acetate	EST	1207	1211		0.1		
(2*E*,4*E*)-Nonadienal	ALD	1216	1210			0.3	0.4
*trans*-Carveol	MO	1221	1215	0.1			
Pulegone	MO	1239	1233	0.4			
(2*E*)-Decenal	ALD	1266	1260			4.9	
Nonanoic acid	FA	1277	1272			1.0	0.3
3,5-Dimethoxytoluene	ARO	1280	1275	6.5	0.1		
(*E*)-Anethole	PP	1286	1282	1.2	0.5		1.2
Safrole	PP	1288	1285	5.0	0.1		
Amyl hexoate	EST	1289	1286			1.0	
2-Undecanone	KE	1294	1294	0.4		0.2	
Tridecane	ALK	1300	1300	0.3	0.1		0.2
Undecanal	ALD	1309	1305				0.2
(2*E*,4*E*)-Decadienal	ALD	1319	1315				0.3
α-longipinene	SH	1355	1350		0.8		
Eugenol	PP	1359	1356	0.2			0.1
2-Methyltridecane	ALK	1367	1360	0.3		0.2	0.3
Longicyclene	SH	1377	1371		0.6		
α-Copaene	SH	1378	1374	0.6	0.4	0.2	0.5
Hexyl hexoate	EST	1388	1384			0.8	
β-Elemene	SH	1394	1389	0.3	0.2		0.2
Sativene	SH	1396	1390		0.6		
Tetradecane	ALK	1400	1400	1.9	0.8	0.3	1.1
Junipene	SH	1405	1404		17.2		0.4
(*E*)-Caryophyllene	SH	1422	1417	4.0	6.2		1.2
β-gurjunene	SH	1435	1431	0.4			
γ-Elemene	SH	1433	1434		1.2	0.2	
Paeonal	ARO	1442	1438	23.5			0.9
(*E*)-β-Farnesene	SH	1450	1454	1.2			
Geranyl acetone	MO	1457	1453	1.3	0.7	0.3	2.5
α-Humulene	SH	1459	1452		3.8		0.6
*ar*-Curcumene	SH	1483	1479		1.2	0.2	0.9
β-Selinene	SH	1486	1489		3.7	0.7	1.8
α-Zingiberene	SH	1495	1493		0.4		
α-Muurolene	SH	1499	1500		0.3		
Pentadecane	ALK	1500	1500	4.6	1.6	0.4	1.9
β-Bisabolene	SH	1509	1505	0.7	0.9		0.6
γ-Cadinene	SH	1516	1513		0.2		
Myristicin	PP	1521	1517	0.6			
β-sesquiphellandrene	SH	1529	1521		0.3		
Selina-3,7(11)-diene	SH	1544	1545		9.0		
Germacrene B	SH	1556	1559		2.4		
(*E*)-Nerolidol	SO	1566	1561		0.1		
3-Methylpentadecane	ALK	1570	1571	0.2			0.4
Caryophyllene oxide	SO	1580	1583	0.2	0.6		0.3
1-Hexadecene	ALKE	1592	1588	0.1			
Cedrol	SO	1593	1600		0.2		0.4
Hexadecane	ALK	1600	1600	0.1	0.4		
Isofuranodiene	SO	1689	1688^e^		12.3		
Heptadecane	ALK	1700	1700	0.3	0.1	0.2	0.9
Phytane	DIT	1790	1795	0.2			0.3
Octadecane	ALK	1800	1800	0.2	0.1		0.6
Hexadecanal	ALD	1819	1815			0.1	
Hexahydrofarnesyl acetone	PA	1840	1845	0.2	0.1	0.1	0.8
Nonadecane	ALK	1900	1900			0.1	0.3
Methyl palmitate	EST	1928	1926	0.4		0.1	0.7
Cembrene	DIT	1939	1937	0.1			
Hexadecanoic acid	FA	1968	1959				2.2
Eicosane	ALK	2000	2000				0.1
Isopropyl hexadecanoate	EST	2028	2023	0.1			
Isopropyl palmitate	EST	2033	2024				0.4
Methyl linoleate	EST	2090	2094	0.2			0.6
Methyl linolenate	EST	2106	2098	0.2		0.1	0.3
Tricosane	ALK	2300	2300				0.1

^a^Compounds are ordered according to their elution from a ZB-5MSI capillary column; ^b^Grouped compounds: ALK, alkanes; ALC, alcohols; ALD, aldehydes; KE, ketons; EST, esters; FA, fatty acids; FUR, furans; ARO, aromatics; ALKE, alkenes; NITR, nitrogen-containing compounds; PA, polyacetylenes; PYR, pyrone; MH, monoterpene hydrocarbons; MO, oxygenated monoterpenes; SH, sesquiterpene hydrocarbons; SO, oxygenated sesquiterpenes; DIT, diterpenes; PP, phenylpropanoids. ^c^Linear retention index (RI) calculated using a mixture of *n*-alkanes. ^d^Literature RIs taken from ADAMS^53^ or NIST 17^54^ libraries. ^e^RI taken from Maggi *et al*.^71^.

In the volatile fraction of *A*. *carmichaelii* we identified 74 components (Table 1). Junipene had the highest relative content (17.2%), followed by isofuranodiene (12.3%) and γ-selinene (9.0%).

The analysis of volatile fraction of *E*. *ulmoides* gave the fewest components (56 VOCs). The most abundant component was hexanal (17.1%), followed by nonanal (11.0%).

The greatest diversity of VOCs (81 types) was detected in *P*. *ternata* (Table 1). Benzeneacetonitrile (syn. benzyl cyanide) had the highest relative content (14.0%), followed by hexanal (13.1%) and nonanal (5.3%) (Table 1).

### Behavioural responses of *L*. *serricorne* to paeonal from *E*. *kansui* VOCs

#### Y-tube olfactometer bioassays

**Figure 3:**
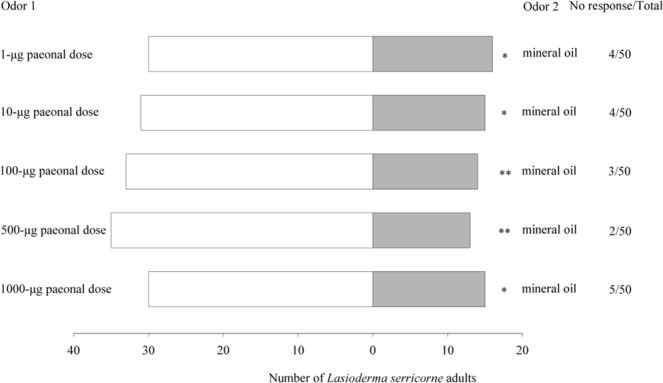
Olfactory responses of *L*. *serricorne* to different doses of paeonal in Y-tube olfactometer tests. Significant differences: *P < 0.05 and **P < 0.01 (chi-square test).

*L*. *serricorne* showed significant relative preferences for paeonal at doses of 1 μg (*χ*^2^ = 4.261, *df = *1, *P* = 0.039), 10 μg (*χ*^2^ = 5.565, *df* = 1, *P* = 0.018), and 1000 μg (*χ*^2^ = 5.000, *df = *1, *P* = 0.025), and highly significant relative preferences at doses of 100 μg (*χ*^2^ = 7.681, *df* = 1, *P* = 0.006) and 500 μg (*χ*^2^ = 10.083, *df* = 1, *P* = 0.001), when compared with mineral oil (Fig. 3).

#### Six-arm olfactometer bioassays

**Figure 4:**
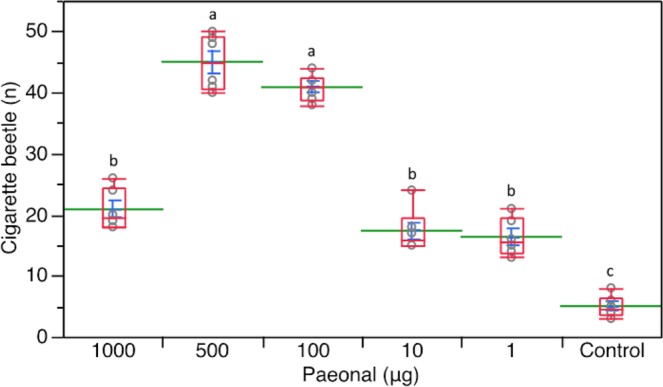
Olfactory responses of *L*. *serricorne* to different doses of paeonal in a six-arm olfactometer assays. Control was mineral oil. Each box plot indicates the median (red line) and its range of dispersion (lower and upper quartiles and outliers); green lines and blue T-bars show means and standard errors, respectively. Above each box plot, different letters indicate significant differences (Wilcoxon test, *P* < 0.05).

In these experiments, all the paeonal doses tested were more attractive than the mineral oil control (Friedman test: *χ*^2^ = 26.722, *df* = 5, *P* < 0.001, Wilcoxon tests: *P* = 0.027–0.028) (Fig. 4). Significantly more *L*. *serricorne* entered the arm connected to the vessel that contained 100- and 500-μg paeonal doses over the arm with 1-, 10-, and 1000-μg doses (Wilcoxon tests: *P* = 0.026–0.027). There was no significant relative difference in attraction between 100- and 500-μg paeonal doses (Wilcoxon test: *P* = 0.136), nor among 1-, 10-, and 1000-μg doses (Wilcoxon test: *P* = 0.115–0.674) (Fig. 4).

## Discussion

In Y-tube olfactometer bioassays, *L*. *serricorne* adults were strongly attracted by VOCs emitted by all CMMs tested in this study, suggesting a strong plasticity of the insect olfactory system in locating suitable food sources. In the Anobiidae family, attraction to a range of CMMs was also reported for the adults of the drugstore beetle, *Stegobium paniceum* (L.), and it is probably related to the high polyphagia of these species^51^.

Furthermore, in our behavioural bioassays, *L*. *serricorne* adults exhibited significantly different preferences among the four CMMs examined, with the ranking *E*. *kansui* > *A*. *carmichaelii* > *E*. *ulmoides* > *P*. *ternata*. Since experiments were carried out in the absence of visual and contact stimuli, this clear ranking preference showed by *L*. *serricorne* adults indicated that plant-borne VOCs provide important cues in the selection of a preferred host by this pest. Thus, the results might partially explain why the damage caused by *L*. *serricorne* differs in degrees depending on the CMMs species involved^57^. Different stored-product pests, such as *Trogoderma granarium* Everts (Coleoptera: Dermestidae), *Tribolium castaneum* Herbst (Coleoptera: Tenebrionidae), *C*. *maculatus*, *S*. *zeamais* and *S*. *paniceum* showed different preferences for the volatiles of several stored products^27,29,51,58^.

Earlier research showed that there were only 13 components identified from the VOCs of *E*. *ulmoides* bark, with the most abundant compound nonanal (17.5%)^59^. However, 56 VOCs were detected in *E*. *ulmoides* bark in the present study, with hexanal (17.1%) as the most abundant compound. This discrepancy may be linked to the differences among experimental protocols used in the two studies, as well as to different growing conditions and harvesting time of *E*. *ulmoides* plant material, which may significantly affect the VOC bouquet, as already noted for other botanical species. Concerning the other three CMMs in our study, GC–MS analysis revealed that *P*. *ternata* had the richest volatile profile with 81 components, followed by *E*. *kansui* and *A*. *carmichaelii*, with 77, and 74 components, respectively. Furthermore, the most abundant compounds varied among the four CMMs species. Thus, the differing olfactory responses of *L*. *serricorne* among CMMs species may be related to differences in both the types and air concentrations of volatile components. However, only a limited number of the VOCs emitted by host plants are crucial for host location by phytophagous insects^60,61^. Herein, paeonal - identified from the preferred CMM *E*. *kansui* - was attractive to *L*. *serricorne* adults at various doses. Furthermore, it showed the highest relative preferences for 100- and 500-μg paeonal doses, indicating that both the types and doses of VOCs from CMMs may influence the beetle olfactory responses^62^. Besides, the attractiveness of junipene, hexanal, benzeneacetonitrile (each was the most abundant compound in one of the other CMMs), or other compounds identified in the study also needs to be further studied using electroantennographic assays, behavioural, and field trapping tests^29,63^. Potential lures could be developed from these tests, which will aid in the development of new safe and effective trapping strategies to monitor and control this pest. Information about the bioactivity of these compounds on other insects are still scarce. However, it is worthy to note that benzeneacetonitrile, identified from the volatiles released by adult mature male *Schistocerca gregaria* Forskal (Orthoptera: Acrididae), had strong repellent effects on gregarious mature males^64^, outlining that the same VOCs can lead to extremely diverse behavioural responses in insects, therefore potential non-target insects arising from the employ of VOCs for monitoring and control should be always considered^65^.

Besides, it has been also showed that an important parasitoid of *L*. *serricorne*, *Lariophagus distinguendus* Forster (Hymenoptera: Pteromalidae)^66^, mainly rely to the VOCs emitted by adult beetle feces when locating its host, stressing that the potential impact of the VOCs proposed for real-world use should be evaluated also on biocontrol agents of the targeted pest, to shed light on potential behavioural changes^67,68^. In this framework, the attractiveness of VOCs from the CMMs to *L*. *serricorne* parasitoids deserves further research. Such information would be useful to boost biocontrol management strategies based on the employ of hymenopteran parasitoids to control *L*. *serricorne* attacking CMMs.

In conclusion, there were three key findings arising from this study. First, Y-tube olfactometer bioassays showed that VOCs from all four CMMs strongly attract *L*. *serricorne* adults. *L*. *serricorne* exhibited a significant preference for *E*. *kansui* VOCs among the four CMMs tested. Second, VOCs differed both in the type and relative content of their components among the CMMs, which may account for the different behavioural responses of *L*. *serricorne* adults to various CMMs. Finally, *L*. *serricorne* showed a significant preference for paeonal tested at various doses, with 100- and-500 μg being the most attractive. These results indicated that this olfactory cue plays an important role for food location in cigarette beetles. From an applied point of view, basic knowledge reported here may be useful – pending proper field evaluation in real-world conditions – to develop novel monitoring and control (e.g., “lure and kill” technique) tools for this pest.

In particular, paeonal identified from *E*. *kansui* VOCs could be exploited alone, as an highly effective lure for both sexes, or in combination with sex pheromone to improve current trapping system for male cigarette beetles^34–36^. Finally, a further intriguing perspective is to use this food-borne VOC attractant to enhance the effectiveness of selected physical cues (UV black light) exploited for monitoring of *L*. *serricorne* beetles^30,69,70^.
